# Supplementary material for: Biomass-Derived Carbon Molecular Sieves Applied to an Enhanced Carbon Capture and Storage Process (e-CCS) for Flue Gas Streams in Shallow Reservoirs
Source: Nanomaterials (Basel). 2020 May 20;10(5):980. doi: 10.3390/nano10050980 (PMC7279535; doi:10.3390/nano10050980)
Supplement: Supplementary file 1 [file nanomaterials-10-00980-s001.pdf]

nanomaterials

Supporting Information

Article

Biomass-Derived Carbon Molecular Sieves Applied to an Enhanced Carbon Capture and Storage Process (e-CCS) for Flue Gas Streams in Shallow Reservoirs

Elizabeth Rodriguez Acevedo [1,2,*], Camilo A. Franco [1], Francisco Carrasco-Marín [3], Agustín F. Pérez-Cadenas [3] and Farid B. Cortés [1,*]

1. Grupo de Investigación en Fenómenos de Superficie–Michael Polanyi, Facultad de Minas, Universidad Nacional de Colombia-Sede Medellín, Medellín 050034, Colombia; caafrancoar@unal.edu.co
2. Grupo de Investigación en Materiales Avanzados y Energía-MATyER, Facultad de Ingeniería, Instituto Tecnológico Metropolitano-ITM, Medellín 050034, Colombia
3. Research Group in Carbon Materials, Faculty of Sciences, University of Granada, 18071 Granada, Spain; fmarin@ugr.es (F.C.-M.); afperez@ugr.es (A.F.P.-C.)

* Correspondence: ecrodrig@unal.edu.co; elizabethrodriguez@itm.edu.co (E.R.A.); fbcortes@unal.edu.co (F.B.C.) Tel.: +57-301-399-5162 (E.R.A.); +57-318-347-4625 (F.B.C.)

Supplementary Information

The TGA analysis for SS-10 and SS-20 presents a variation of impregnation percentage. The real impregnation percentages are calculated after 100 °C and correspond to 8.6 and 20.8 %, respectively. The real impregnation percentages are calculated after 100 °C. At this temperature the humidity is eliminated, and the subsequent weight loss is due the carbon oxidation. The Figure S1 presents the thermogravimetric results.

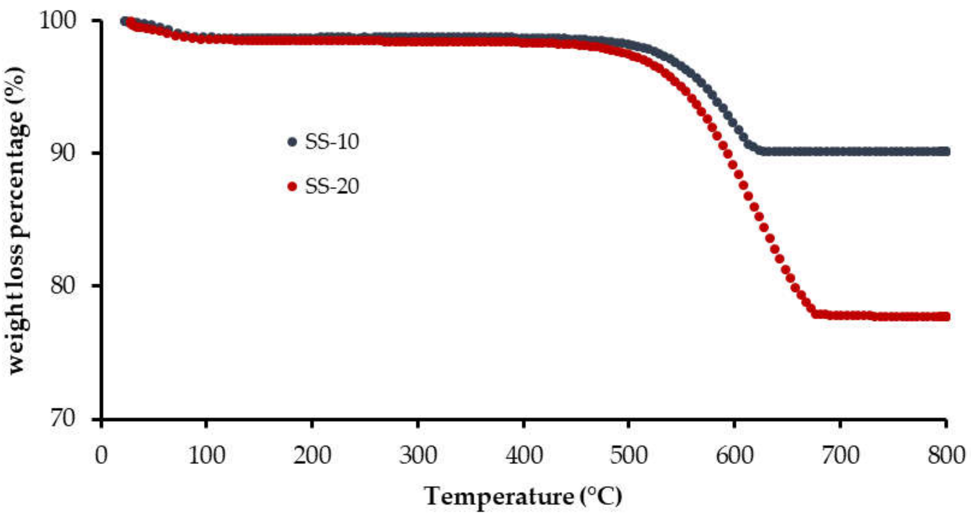

Figure S1. Thermogravimetric analysis for SS-10 and SS-20.
